# De-implementing and sustaining an intervention to eliminate nursing home resident bed and chair alarms: interviews on leadership and staff perspectives

**DOI:** 10.1186/s43058-021-00195-w

**Published:** 2021-08-24

**Authors:** Christine W. Hartmann, Christopher Gillespie, George G. Sayre, A. Lynn Snow

**Affiliations:** 1 VA Bedford Healthcare System, 200 Springs Road (152), Bedford, MA 01730 USA; 2 University of Massachusetts Lowell Department of Public Health, 220 Pawtucket St, Lowell, MA 01854 USA; 3 VA Puget Sound Health Care System, 9600 Veterans Dr SW, Tacoma, WA 98498 USA; 4 The University of Washington Department of Health Services, 1959 NE Pacific St, Seattle, WA 98195 USA; 5 Tuscaloosa VA Medical Center, 3701 Loop Rd, Tuscaloosa, AL 35404 USA; 6 The University of Alabama Department of Psychology, 745 Hackberry Ln, Tuscaloosa, AL 35401 USA; 7 The University of Alabama, Alabama Institute on Aging, 745 Hackberry Ln, Tuscaloosa, AL 35401 USA

**Keywords:** Nursing homes, Long-term care, Qualitative research, Interview, Implementation science, United States Department of Veterans Affairs

## Abstract

**Background:**

Improving nursing home quality of care relies partly on reducing or stopping ineffective or harmful practices, a process known as de-implementation. We know little about de-implementation in this setting. Relatively recent policy changes reclassified resident position-change (bed and chair) alarms, which monitor resident movement, as restraints. This created an optimal environment in which to study impressions of an alarm de-implementation and sustainment intervention.

**Methods:**

This cross-sectional interview study focused on understanding participants’ experience of a quality improvement program in the Department of Veterans Affairs Community Living Centers (nursing homes). The program’s goal was to improve resident outcomes and staff communication and teamwork through, among other foci, eliminating resident position-change alarms. The Community Living Centers were located in geographically dispersed areas of the continental United States. Interview participants were leadership and staff members from seven Community Living Centers. We conducted in-depth, semi-structured qualitative interviews using a convenience sample and used a thematic analytic approach.

**Results:**

We conducted seventeen interviews. We identified five main themes: *Initiating De-implementation* (compelling participants with evidence, engaging local leadership, and site-level education and training), *Changing Expectations* (educating staff and family members), *Using Contrasting Approaches* (gradual or abrupt elimination of alarms), *Witnessing Positive Effects of De-implementation* (reduction in resident falls, improved resident sleep, reduction in distressing behaviors, and increased resident engagement), and *Staying the Course* (sustainment of the initiative).

**Conclusions:**

Findings highlight how participants overcame barriers and successfully eliminated resident position-change alarms and sustained the de-implementation through using convincing evidence for the initiative, local leadership involvement and support, and staff and family member education and engagement. These findings and the resulting three-phase process to support nursing homes' de-implementation efforts expand the de-implementation science knowledge base and provide a promising framework for other nursing home-based de-implementation initiatives.

**Supplementary Information:**

The online version contains supplementary material available at 10.1186/s43058-021-00195-w.

Contributions to the literature What is already known about the topic?

- Improving healthcare sometimes involves stopping or reducing services or practices known to be ineffective or harmful (de-implementation), but evidence about how best to approach de-implementation in nursing homes is sparse.
- Findings from our interviews about nursing home staff members’ efforts to eliminate resident bed and chair alarms indicate the importance of presenting compelling evidence regarding the de-implementation effort and of having local leadership convinced of the importance of the change.
- Based on the results, we propose a potential three-phase process for nursing home de-implementation: prepare for de-implementation, facilitate the process and evaluation of de-implementation, and evaluate and sustain.

## Background

Improving healthcare sometimes involves stopping or reducing services or practices known to be ineffective or harmful. This process, known as de-implementation, has potential public health benefits as well as implications for healthcare waste reduction [1]. We understand less about de-implementing existing practices than we know about implementing new ones [2–4], although we do know successful de-implementation may rely on different structures and processes than successful implementation [1, 5]. Norton and Chambers, for example, highlight the unique importance for de-implementation of removing, replacing, reducing, and restricting treatments or services. In nursing homes, de-implementation may be particularly important because nursing home quality is measured in part by reductions in and removals of things such as restraints, indwelling urinary catheters, and inappropriate antipsychotic medications. But evidence about how best to approach de-implementation in nursing homes is sparse [6–8].

Recently, de-implementing the use of nursing home resident position-change alarms that monitor resident movement (e.g., bed and chair alarms) has received attention. Results from studies increasingly point to the ineffectiveness of these alarms at stopping falls and poor outcomes for residents [9–14]. Effective November 2017, Centers for Medicare and Medicaid Services’ revisions to the State Operations Manual therefore classified these alarms as restraints. The Department of Veterans Affairs (VA) reclassified position-change alarms as restraints in October 2017 to meet the Centers for Medicare and Medicaid Services’ November 2017 start date. This change in VA policy created optimal circumstances to study the de-implementation of this nursing home practice.

We implemented a multi-component, evidence-based quality improvement program in select VA nursing homes, which are known as Community Living Centers (CLCs). Investigating CLC leadership and staff impressions of alarm de-implementation in the VA, the nation’s largest integrated health system, provided an opportunity to study nursing homes that were potentially still implementing the practice within an environment supportive of de-implementation.

## Methods

### Program overview

Leaders from VA’s national facility-based care team in Washington, D.C. selected eight CLCs to take part in a quality improvement learning intensive aimed at improving resident outcomes on Centers for Medicare and Medicaid Services quality measures and staff communication and teamwork processes through a structured, evidence-based curriculum that built on the outcomes of a series of research studies and quality improvement projects [15–17]. The CLCs were selected based on their historically low performance on VA’s quality measure star rating (one star lowest and five highest), which is based on the Centers for Medicare and Medicaid Services methodology that uses fifteen measures. At the time of the learning intensive, the VA’s quality measure rating used eleven of the Centers for Medicare and Medicaid Services quality measures, including restraint use, combining data from a CLC’s four most recent quarters. When the learning intensive began, all participating CLCs were either one or two stars on the quality measure. The learning intensive followed the Institute for Healthcare Improvement’s [18] guide for conducting a breakthrough series collaborative, which enables organizations to make improvements in quality by learning from each other as well as from experts. It comprised two in-person and three virtual learning sessions interspersed with action periods over the course of a 9-month learning intensive (August 2018 to April 2019). VA national facility-based care leadership team members attended each of the sessions. Each CLC also received two site visits from program staff during the middle of the learning intensive to support the CLCs’ efforts.

Each participating CLC created a local leadership team that took part in the in-person and virtual learning sessions, led the local intervention implementation during the action periods between sessions, and organized the local site visits. Additional local CLC staff were invited to attend the virtual learning sessions and site visits at each CLC’s discretion. The local CLC leadership team comprised the medical director, nurse leader, resident assessment coordinator for the Minimum Data Set, and the quality manager associated with the CLC.

Content for the learning intensive focused on improving care quality through improved staff communication and teamwork processes. Alarm reduction was introduced as part of the emphasis on the importance of residents’ sleep. An initial in-person learning session introducing the importance of creating high-functioning, relationship-based teams. The second in-person learning session built on the first and introduced using individualized care and high staff engagement to eliminate alarms, prevent falls, and protect sleep. The de-implementation of resident bed and chair alarms and sustainment of this de-implementation became a key focus for participants after this learning session. The third learning session was virtual and focused on a consultation with a geriatric psychiatrist about individualizing care for residents with distress behaviors to reduce stress for staff and residents; alarm reduction was mentioned when applicable to a CLC’s experience. The fourth learning session was also virtual and guided participants in how to choose and implement a 1-month performance improvement project of their own choosing (not necessarily alarm reduction), using the principals learned in the intensive. The final session was a virtual outcomes congress where participants shared their experiences and outcomes across sites.

### Interview participants and recruitment

Immediately following the end of the learning intensive, in May to July 2019, we recruited a convenience sample from the population of local leadership team members and staff who participated in the CLCs’ alarm de-implementation efforts (total population *n* = 93). We contacted all 93 potential participants through email, setting up a telephone interview with individuals who indicated willingness to participate in in-depth, semi-structured individual interviews. Interviews were conducted by a male Ph.D.-level researcher (C.G.) with extensive training in qualitative research methods. Interviews lasted 20 to 30 min. The interviewer and participants had no prior relationship. Participants were informed about the goals of the study during recruitment and again prior to the beginning of the interview. At the beginning of each interview, the interviewer introduced himself and described the nature of the interview and the medical facility at which he worked.

### Interview guide and interview process

We designed an interview guide to explore participants’ experiences with the learning intensive, their understanding of the evidence presented regarding the use of resident bed and chair alarms, and their efforts at eliminating or reducing the use of these alarms (see Additional file 1). First, researchers experienced in qualitative interviewing (C.H., C.G., and L.S.) consulted with members of the learning intensive team to understand the type and format of material presented. These researchers then wrote specific questions relating to alarm de-implementation, creating an interview guide. They revised questions iteratively throughout the data collection process, as the need to rephrase questions became apparent or as preliminary analyses identified themes that we wished to explore further. For example, as local leadership indicated that staff occasionally requested the reintroduction of alarms, questions about this were included in subsequent interviews.

We offered participants as much time as they wanted for the interviews and used the semi-structured interview guide throughout to elicit details and rich descriptions. All interviews ended with an open-ended question, enabling participants to provide any additional information or address salient topics the interview guide may have missed.

### Data analysis

Interviews were recorded and transcribed verbatim to facilitate qualitative data analysis, which was conducted using Atlas.ti v. 7. A Ph.D.-level qualitative researcher (C.G.) analyzed the data following a thematic analytic approach consisting of the identification of salient themes [19, 20].

The analyst coded transcripts to identify passages indicating conceptually distinct ideas related to alarm de-implementation. Passages associated with each code were extracted and reviewed to develop a codebook consisting of codes and their various uses. This enabled development of summary statements with maximum fidelity to participants’ reported perceptions and experiences. When necessary, transcripts were examined to identify alternative ways to form summaries. The analyst used conceptual memos to guide analysis and describe initial themes. Through successive rounds of coding and memo writing, distinct themes were identified and scrutinized for consistency through comparison to possible counter examples. Summaries of thematic elements were then integrated to represent participants’ experiences.

Initial themes were the use of evidence in making decisions regarding the use of alarms, efforts to educate CLC staff about discontinuing the use of alarms, local approaches to de-implementation, effects of de-implementation on staff and residents, and the sustainability of de-implementation efforts. The full research team (C.H., C.G., G.S., and L.S.) reviewed the preliminary analysis and supporting transcript data and held multiple discussions about the emerging themes, using data from the transcripts to ground the discussions. In the final analytic stages, the full research team revised themes and parsed final themes to a finer-grain level.

This interview project was sanctioned as quality improvement and exempt from Institutional Review Board oversight in accordance with the Department of Veterans Affairs, Office of Research and Development Program Guide 1200.21 [21]. Employee unions reviewed and approved the interview guide prior to its use. This manuscript follows the Consolidated Criteria for Reporting Qualitative Studies (see Additional file 2).

## Results

We received positive responses from seventeen (18%) potential participants, representing a convenience sample of participants from seven of the eight CLCs. We completed interviews with all seventeen participants. Fourteen interviews were with local leadership team members from seven CLCs. These were Chief Nurses, Nurse Managers, and Registered Nurses (RNs) serving as Quality Management Specialists. An additional three staff members from three different CLCs agreed to participate in interviews. These staff members did not participate in the in-person portions of the learning intensive but did participate in the virtual training and program implementation and thus knew about or were involved in resident bed and chair alarm de-implementation and sustainment efforts. These were two Certified Nursing Assistants (CNAs) and a Nurse Manager (see Table 1).

**Table 1:** Interview participant role and status of resident bed and chair alarm de-implementation by site

Site	Leadership team participant job title (interview identification number)	Staff participant Job title (interview identification number)	Status of alarm de-implementation during learning intensive
A	Chief Nurse (1) Quality Management Specialist (3) RN, MDS Coordinator (4)	CNA (89) CNA (92)	De-implementing and sustaining
B	RN, Quality Management Specialist (7) RN (8)		Sustaining
C	MD (9)	RN, Nurse Manager (74)	Sustaining
D	Quality Management Specialist (15)		Sustaining
E	Chief Nurse (17) MD (18) Quality Management Specialist (19) RN, MDS Coordinator (20)		De-implementing and sustaining
F	RN, Quality Management Specialist (23) RN, MDS Coordinator (24)		De-implementing and sustaining
G	RN, Nurse Manager (29)		De-implementing and sustaining
***N*= 17**	**14**	**3**	

We identified five themes within the de-implementation process: *Initiating De-implementation* (compelling participants with evidence, engaging local leadership, and site-level education and training), *Changing Expectations* (educating staff and family members), *Using Contrasting Approaches* (gradual elimination of alarms or abrupt elimination of alarms), *Witnessing Positive Effects of De-implementation* in falls, improved sleep, reduction in distressing behaviors, and increased resident engagement), and *Staying the Course* (sustainment of the initiative).

As indicated in Table 1, some participating CLCs had already de-implemented bed and chair alarms by the time of the learning intensive, so their efforts focused exclusively on sustainment.

In the quotations we present, we removed all identifying information and changed any reference to a “patient” to “[resident],” in keeping with a person-centered approach.

### Initiating de-implementation

Although CLCs were encouraged to de-implement of alarms, each site had the autonomy to initiate and sustain discontinuation of alarms. Participants indicated that the evidence presented during the in-person learning session was critical to fostering their intent to initiate site-level de-implementation efforts. They identified engagement of local leadership champions and local site-level education as the two critical factors in moving from intention to act to actual implementation.

#### Compelling evidence

Evidence shared in the learning intensive was critical to participants’ intention to initiate and sustain the de-implementation of alarms:

There’s evidence that the alarm does not actually prevent falls. [We learned] all the alarm does is keep our [residents] up at night, and they’re not getting enough sleep. So we provided education for all of the nursing staff and bedside staff. That’s when we started taking the alarms off and increasing our engagement [with] and oversight of the [residents]. (17 – site E, Chief Nurse)

In particular, participants responded to research indicating alarms were ineffective in preventing falls and could even *result in* falls:

What they were teaching us was that the alarms, actually do cause falls, because [the noise] startles people, it scares some people…. If the alarm is going off exactly where a fall occurred, then that alarm is causing them to fall. It’s not even necessarily the veteran or [resident] who has the alarm on. It could’ve been their neighbor next door or their neighbor across the hall. (15 - site D, Quality Management Specialist)

One described the learning session structure as critical to uptake:

[The instructors shared] a lot [of] the reasons behind it. Otherwise, if we had never been to the learning intensive, I’m guessing we never would’ve pursued [eliminating alarms] at all….That way we can come back to the [CLC] and disseminate the information with a good, solid base. (23 – site F, RN, Quality Management Specialist)

#### Engaging local leadership

A critical factor in implementing de-implementation was achieving buy-in from CLC leaders who had the authority to make decisions about the use of alarms and could facilitate their removal. Chief Nurses, Nurse Managers, and Quality Management Specialists were critical to making these decisions in the CLCs:

The Nurse Executive was saying, “By this date, by this time, I want alarms gone. So we’re going to work on this and we’re going to really push forward.” And our Nurse Managers were pretty great about it. Most of them were like, “This is the movement in long-term care anyway.”… It was kind of easier for them and then for them to facilitate that and spread it throughout their staff. (19 – site E, Quality Management Specialist)

Sometimes it was important for resident-based decision-making groups to be involved in this process as well. “We presented it in a resident council meeting and let the residents know. We presented it to staff and did an education to the staff. And we discontinued it. It wasn’t that long.” (74 - site C, RN, Nurse Manager)

#### Site-level education and training

Upon return to their CLC from the in-person learning session that focused on alarm elimination, the local leadership team shared information with staff. In those CLCs just beginning the process of eliminating alarms, this helped staff understand the importance of de-implementation and the evidence supporting alarms’ lack of effectiveness and provided an opportunity for leadership to address staff concerns:

[The Chief Nurse] did some sessions with the staff, with all of the staff…. I wasn’t present, but she did discuss the reason behind eliminating [alarms]. She shared the data that we were presented with. That was pretty much a vent session for them, for them to say they were concerned, can you tell us why this is happening. It was that type of thing. (15 - site D, Quality Management Specialist)

In-person dissemination of the evidence presented at the learning intensive was an effective strategy for securing staff buy-in:

It was a process of just going from neighborhood to neighborhood, just explaining what we had learned at the conference. And getting buy in from staff. Nurse Managers were key; the doctors were key. It was the real key players that really helped through the process. (24 – site F, RN, Minimum Data Set Coordinator)

### Changing expectations

#### Educating staff members

Despite the on-site presentation of evidence by local leadership, some participants reported continuing reluctance to de-implementing.

Participant: I think we should be allowed to use them again. I think they are very helpful as well. They can prevent a fall, because sometimes you can get to the room fast enough to prevent a fall. (92 - site A, Nursing Assistant) Interviewer: Are there times that an alarm has been used since you’ve been there? Where somebody felt like it was necessary? Participant: Yes …maybe short-term, if you have to step away to go get something, you’d rather know that the person has some form of security than none at all. (89 - site A, CNA)

Some participants noted staff sometimes felt alarms were integral to their CLCs’ workflow. These participants spoke about not merely in terms of a change in practice but a change in culture.

So the staff were a little bit resistant because they were dependent on the use of alarms. It’s like they wait for the alarm to tell them that the [resident] is falling, … It’s a cultural change. All of these years, they were dependent on the alarm to tell them that the [resident] is falling. It’s like taking something away from them. (17 - site E, Chief Nurse)

#### Educating family members

Some participants also indicated it was often necessary to share information about de-implementing alarms with family members who spoke about concerns that eliminating alarms would lead their loved one to fall:

The same information we provided for the staff, we provided… [to] two family members that requested [it]. We provided the same information and gave them records, the fall records of their loved ones, and how our rates generally have decreased. So we showed them evidence that the implementation of no alarms has really been beneficial to their loved ones and to the [resident] population generally. So they saw it and they were okay with it. (17 - site E, Chief Nurse)

### Using contrasting approaches

#### Gradual elimination of alarms or piloting

In some CLCs, alarms were phased out gradually. The first step for this was sometimes an assessment of the relationship between alarms and poor resident outcomes:

The first step was really nice. I got [a layout of the CLC], and then I called the Nurse Manager to obtain the [residents] that have the alarms. Based on that, we wanted to see any related falls in those particular rooms. And we were able to confirm with the information that we obtained at the learning intensive, that it was true: [in] all of the corridors that had bed alarms, there had been multiple fall incidents. (23 - site F, RN, Quality Management Specialist)

Other CLCs piloted the elimination of alarms by beginning with one or two neighborhoods before rolling it out to additional units:

We piloted it in one of the big units, our long-term care unit. We first took it off of two [residents], and we increased the monitoring… We usually put on the alarm because we don’t want the [resident] to fall, but we found that even by the time the alarm goes off, most of the time the [resident] is already on the floor. So we started little, and we went weekly to all of the [residents] in one unit. Then we spread it to the next unit, and the next unit, until we got rid of all of the alarms on all 6 units. (17 - site E, Chief Nurse)

#### Abrupt elimination of alarms

In other CLCs, the alarm de-implementation happened much more rapidly, with alarms being completely eliminated by a particular date throughout all neighborhoods:

Our Chief Nurse [eliminated alarms] based on the realization that these things are irritating, not only to the person that’s sitting on it. They’re irritating to the other people around us. It’s startling. If we can’t have conversations, neither can the residents. And it wasn’t helping to get their needs met. So she gave a date and said, “At this point in time we aren’t going to use these anymore.” (7 - site B, RN, Quality Management Specialist)

One participant noted that their CLC had initially gradually de-implemented alarms, before later deciding to remove them all at once. This change was described as a group effort in response to staff pushback.

[Someone said,] “Why don’t we just take them all at one time?’And when she said that, we thought, "Shoot, that’s a good idea. Why are we doing this the way we’re doing it? Let’s do that." So we ended up just taking them all away. And believe it or not, like I said, a week or two later they were saying, "Wow, this is really working.” (8 - site B, RN)

#### No alarms on site

In many cases, alarms were physically removed from the CLC after de-implementation and were no longer available, thus making it impossible for staff to re-implement their use. In some cases, this was due to the effort of one individual:

I looked for every alarm I could find, that they ever had used, and took them out of the building. That way we’re not susceptible to, “Maybe we need to use one of those. She’s [the nurse manager’s] not here.” That kind of thing. No, there’s no alarms in my building. (29 - site G, RN, Nurse Manager)

### Witnessing positive effects of de-implementation

#### General improvement for residents

Participants viewed elimination of alarms as a critical part of the learning intensive’s larger bundle of practices implemented to improve resident quality of life.

It’s hard to correlate residents’ behavior with alarms, because there are a couple of different initiatives that we started: being proactive, talking to the residents’, looking into their sleep, doing deep dives about the issues related to sleep. If they want to get up a little later, [we] let them sleep. To take the alarms off [was also an initiative]. I mean, overall, we see that the veterans [residents] are happier. There are less altercations. (18 - site E, Doctor of Medicine)

Seeing the positive effects of improved sleep on the residents’ quality of life was profoundly persuasive in leading staff to embrace and sustain the de-implementation of alarms.

When we found out how important it is for our veterans [residents] to have enough sleep for them to function during the day, that…really motivated us to get rid of the alarms.” (17 - site E, Chief Nurse)

#### Reduction in falls

CLC local leadership indicated eliminating alarms led to a reduction in the frequency of resident falls. Participants noted this, as exemplified in this quote: “Absolutely, yes. We’ve seen a decrease in falls with major injury.” (23 - site F, RN, Quality Management Specialist).

#### Reduction in disruptive behavior

Improved sleep was seen as leading to a decrease in residents’ distressing behaviors.

I also look at disruptive behaviors as well, and we’ve had a decrease in those. And I know that was one of the reasons behind allowing them to have this uninterrupted sleep, that it helps with their behaviors as well. So we’ve also seen a decrease in that, in addition to the falls. (15 - site D, Quality Management Specialist)

#### Resident engagement

Participants also reported that residents getting better sleep enabled residents to better participate in meaningful conversations and activities throughout the day:

I think they’re [residents are] able to have more meaningful conversation. You can call it relationships. A lot of them likely don’t remember it from one day to the next, but in the immediate moment, their life is more pleasant. You can carry on a conversation. (7 - site B, RN, Quality Management Specialist)

In addition to the capacity for meaningful conversations with residents, CLC staff also noted improved sleep led to greater resident responsiveness during the day:

I think it’s been very positive, because along with the noise reduction, we’re working to improve sleep, right? So not having the alarm ringing all day and night [helps], especially during the night, so that the [residents] can be more calm, more responsive to activities. (23 - site F, RN, Quality Management Specialist)

### Staying the course

CLC local leadership received some requests from staff to reintroduce alarms, sometimes temporarily or on a case-by-case basis. These leaders emphasized the importance of being consistent in sustaining alarm elimination, using guidelines to motivate staff and presenting further evidence of alarms’ inappropriateness.

You stay the course and you keep educating. You keep talking. You keep showing them [staff] statistical data, and you keep telling them that we’ve had less falls. And they can’t argue with that. You have to stay vigilant, keep talking, keep educating. You can’t stop. It is a constant, every day, every day. And I haven’t stopped yet. We’re still talking about alarms. I had someone who came back the other day who sent me a message that we needed to put alarms back out, that they needed alarms. (29 - site G, RN, Nurse Manager)

Other participants described a more complete change in culture, one in which alarms were seen as a thing of the past.

I don’t believe we have any intention of going back, and I don’t think staff think of it anymore. It’s just the way we did things when we didn’t know better. (7 - site B, RN, Quality Management Specialist)

## Discussion

Findings from our interviews about CLCs’ efforts to eliminate resident bed and chair alarms highlight potential applications for similar nursing home-based de-implementation efforts. They also expand our general understanding of de-implementation dynamics, particularly regarding processes involving the removal of devices. And they add to the de-implementation knowledge base around the issue of sustainment, where studies to date have devoted limited attention [22, 23].

### Potential applications for similar nursing home-based de-implementation efforts

Lessons learned from participating CLCs’ focus on eliminating alarms are likely generalizable to similar nursing home efforts. The compelling nature of the evidence and having local leadership convinced of the importance of the change were the main factors influencing successful de-implementation. A key facilitator was having individuals with decision-making authority attend the learning intensive, as those individuals were then able to lead the initiative in their local CLCs. This is consistent with Crogan and Dupler’s [24] experience when implementing an alarm elimination program in a nursing home that initially struggled with de-implementation. Similar to our work, they designed an educational program for staff that addressed the safety issues around alarm use and taught staff how to assess and intervene to de-implement. The director of nursing and other site leadership led the intervention, which was successful at eliminating all alarms in 5 months without an increase in falls. Bressler et al. [10], who successfully implemented a program to eliminate position-change alarms in a home for residents with Alzheimer’s disease or advanced dementia, took a similar approach, also with a leadership-driven program and education using compelling evidence. Our program’s design provides another meaningful template for successful alarm elimination.

Two of the interviews cited in this paper mentioned spreading the alarm de-implementation program from unit to unit across the entire nursing home. We believe this was probably a direct result of their using the methods we taught in our program, which emphasize piloting all change efforts in small increments. We taught participants to start with one unit, pilot their intervention, monitor and learn from the pilot project, and adapt as necessary before spreading to another unit and repeating this cycle. As a Chief Nurse said, “We started little…Then we spread it to the next unit, and the next unit.” This is in line with literature emphasizing the importance of keeping change manageable [25] and provides a pragmatic model for spreading quality improvement interventions.

An issue that emerged for some of our participants during de-implementation and sustainment was an entrenched belief on the part of staff and family members that alarms help keep residents safe. Participants addressed this through continued education and steadfast maintenance of a “no alarm” stance. Crogan et al. [24] and Bressler et al. [10] also noted some staff and family member reluctance and the importance of including them in the elimination process, mainly through education and engagement. This pressure from staff to continue to use or bring back alarms illustrates a challenge of de-implementation and sustainment and highlights the importance of strategies to remind current and future staff of why the change was made and what has been gained. To address this, our program taught participants about the importance of metacognition [26]. Through experiential exercises, they learned the value of explicitly labeling gains as they occur and referring to them going forward to internalize (rather than overlook) evidence of a program’s positive effects. Our findings indicate participants’ de-implementation and sustainment efforts were successful in part due to local leadership implementing these metacognition techniques. They may be valuable to nursing homes involved in similar initiatives.

### Expanding understanding of de-implementation

Sauro et al. [27], in a hospital-based study comparing the implementation of a high-value practice and the de-implementation of a low-value one, found clinical leader preferences were the greatest barrier to de-implementation; on-site education was the greatest facilitator. Our findings regarding the importance of engaging and supporting local leadership as change agents and providing not only educational materials but also education techniques (metacognition) expand this understanding. This may also help researchers as well as quality improvement interventionists understand where to focus practice change efforts when using checklists or other generic tools. Flottorp et al. [28], for example, conducted a systematic review and developed a 57-item checklist listing determinants of healthcare professionals’ practice grouped under seven domains. As Flottorp et al. [28] themselves suggest, their list is likely too long to be of use in quality improvement projects. But triangulating their domains with our results, we find that checklist items from three domains might be most relevant in the case of de-implementing alarms or other devices in nursing homes: “guideline factors” (strength, applicability, and other factors regarding evidence for de-implementation), “individual health professional factors” (knowledge, skills, attitudes, and behavior), and “patient factors” (including needs and beliefs—in the nursing home setting this should be expanded to include family member factors). Using findings from de-implementation studies such as ours to prioritize their domains to those of main import is one way qualitative findings such as ours can expand the practical impact of a more general knowledge base.

Consistent with the definition of sustainability as a process, not an outcome [28], CLCs participating in our program achieved sustainability through a variety of methods, including education of staff and local leadership’s consistent commitment to the value of the effort. These findings are congruent with the literature on what facilitates sustainment, such as having leaders and champions, training, and stakeholder engagement [28]. The case of eliminating alarms may differ from some other examples of de-implementation, in that physically removing alarms is possible and there are no other devices that staff can directly substitute. This potentially made sustainment easier. With no alarms at hand, staff could not use them, and leadership could use the absence of the physical device as a way to navigate frontline staff requests for occasional use. This differs, for example, from efforts to de-implement antipsychotic medications, where some evidence points to unintended increases in the use of other medications [29, 30].

Shelton et al.’s [31] review of sustainability in public health and healthcare identified program evaluation as another factor influencing sustainability, although that review also indicated that very little is known about the nature and influence of program evaluation on sustainability. Our participants’ comments on the importance of evaluation to their sustainment efforts thus shed needed light in this area. This also contributes to a potential modification of Niven et al.’s [22] de-implementation framework, which posits four de-implementation phases: identify low-value clinical practices, facilitate the de-implementation process, evaluate the de-implementation outcomes, and sustain de-implementation. Based on our findings, we can modify Niven et al.’s framework into three phases: prepare for de-implementation, facilitate the process and evaluation of de-implementation, and evaluate and sustain. In our findings, CLCs found evaluation to be important for both de-implementation *and* sustainment. At least one CLC, for example, did an initial evaluation of the state of alarm use and falls before eliminating alarms. And a number of CLCs incorporated evaluation into their sustainment efforts, using the data they collected to identify the continued benefits of alarm elimination. Evaluation thus exerted a positive influence when intertwined with both phases, instead of existing as a separate phase. This is congruent with the procedures outlined by Crogan et al. [24], who also conducted an evaluation before de-implementing. We therefore propose our adapted three-phase framework as a potential change to Niven et al.’s original work.

This interview study was cross-sectional and included only seven CLCs. But there was consistency of findings across sites and agreement with existing literature. While the study was conducted within VA, the overarching policy environment regarding alarm use in VA is similar to that of the non-VA environment, increasing the likelihood the findings can be applied outside the VA setting. Participants in the study were mostly CLC local leadership team members; we had few frontline staff participants, and no residents were interviewed, which is a limitation.

Our findings describe sustainability at varying points, as some CLCs had already eliminated alarms before participating in the learning intensive while others de-implemented and sustained during the learning intensive. We document each CLC’s status in Table 1 but did not break the analysis apart by CLC. Still, we believe our ability to describe sustainment representing different lengths of time is a strength of our work. We acknowledge that CLC participants probably felt a push from VA national facility-based care leadership to adopt all aspects of the learning intensive program, including alarm de-implementation and sustainment. This push may have felt particularly strong because they were selected to participate in the program due to their low performance. This may have influenced uptake and sustainment beyond the facilitators highlighted in the results and may make the results less generalizable. The push from VA leadership, however, took place within a national cultural shift away from alarms, as demonstrated by the 2017 Centers for Medicare and Medicaid Services reclassification of position-change alarms as restraints. We therefore believe our results are likely still generalizable, including to nursing homes outside the VA.

## Conclusions

Participants in this quality improvement program successfully de-implemented resident position-change alarms. Consistent with the literature, our findings supported the importance of the following facilitators of change: convincing evidence for the initiative, local leadership involvement and support, and staff and family member education and engagement. Our findings also expand our understanding of the important, intertwined role of evaluation in de-implementation and sustainment. The successful aspects of this evidence-based de-implementation program, including its learning intensive structure, should be considered for use with other nursing home de-implementation initiatives.

## Supplementary Information

**Additional file 1.** Final interview guide.

**Additional file 2.** Consolidated criteria for reporting qualitative studies (COREQ): 32-item checklist.

## Data Availability

The datasets used and/or analyzed during the current study are available in de-identified form from the corresponding author on reasonable request.

## Abbreviations

CLC: Community Living Center
CNA: Certified Nursing Assistant
RN: Registered Nurse
VA: Department of Veterans Affairs
