# Supplementary material for: De-implementing and sustaining an intervention to eliminate nursing home resident bed and chair alarms: interviews on leadership and staff perspectives
Source: Implement Sci Commun. 2021 Aug 24;2:91. doi: 10.1186/s43058-021-00195-w (PMC8383405; doi:10.1186/s43058-021-00195-w)
Supplement: Supplementary file 1 — Additional file 1. Final interview guide. [file 43058_2021_195_MOESM1_ESM.docx]

**Appendix A: Interview Guide**

**De-Implementing Antipsychotics and Implementing Behavioral Interventions (DIAMOND)**

1. What is your current position?

-[If needed] What are your main responsibilities?

-[If needed] When did you start this role?

2. Please share with me your impressions of the Learning Intensive.

3. How did the Learning Intensive influence your efforts to eliminate, or reduce, the use of alarms?

4. In your efforts to reduce/eliminate the use of [alarms/practices that disrupt sleep], what has been most useful in making progress toward these goals?

5. Which areas have been most difficult to reduce/eliminate, or most resistant to change?

6. What has made these changes difficult?

7. How sustainable to you think the changes you have noticed will be?

8. Which elements do you think might be most difficult to sustain?

9. What additional assistance would be helpful in continuing to address these areas?

10. What changes in resident behavior, engagement, and quality of life have you noticed as a result of your efforts to reduce/eliminate alarms?

11. How has staff behavior, engagement, and quality of life changed?

12. What quality improvement systems, practices, or other structures have been implemented, or improved, since the LI?

13. Has performance in quality measures, or other metrics improved since the LI? Please explain.

14. Do you have any questions for us, or is there anything else you would like to add?
