# Supplementary material for: De-implementing and sustaining an intervention to eliminate nursing home resident bed and chair alarms: interviews on leadership and staff perspectives
Source: Implement Sci Commun. 2021 Aug 24;2:91. doi: 10.1186/s43058-021-00195-w (PMC8383405; doi:10.1186/s43058-021-00195-w)
Supplement: Supplementary file 2 — Additional file 2. Consolidated criteria for reporting qualitative studies (COREQ): 32-item checklist. [file 43058_2021_195_MOESM2_ESM.docx]

**Table**

Consolidated criteria for reporting qualitative studies (COREQ): 32-item checklist

| **No** | **Item** | **Guide questions/description** |
| --- | --- | --- |
| **Domain 1: Research team and reflexivity** |  |  |
| Personal Characteristics |  | These are in the Interview Participants and Recruitment section |
| 1. | Interviewer/facilitator | Which author/s conducted the interview or focus group? |
| 2. | Credentials | What were the researcher's credentials? *E.g. PhD, MD* |
| 3. | Occupation | What was their occupation at the time of the study? |
| 4. | Gender | Was the researcher male or female? |
| 5. | Experience and training | What experience or training did the researcher have? |
| Relationship with participants |  | These are in the Interview Participants and Recruitment section |
| 6. | Relationship established | Was a relationship established prior to study commencement? |
| 7. | Participant knowledge of the interviewer | What did the participants know about the researcher? e*.g. personal goals, reasons for doing the research* |
| 8. | Interviewer characteristics | What characteristics were reported about the interviewer/facilitator? e.g. *Bias, assumptions, reasons and interests in the research topic* |
| **Domain 2: study design** |  | See notes for each item |
| Theoretical framework |  | We describe this as thematic analysis, in the Analysis section |
| 9. | Methodological orientation and Theory | What methodological orientation was stated to underpin the study? *e.g. grounded theory, discourse analysis, ethnography, phenomenology, content analysis* |
| Participant selection |  | These details are in the Interview Participants and Recruitment section and Table 1 |
| 10. | Sampling | How were participants selected? *e.g. purposive, convenience, consecutive, snowball* |
| 11. | Method of approach | How were participants approached? e*.g. face-to-face, telephone, mail, email* |
| 12. | Sample size | How many participants were in the study? Included in Participants and Recruitment section |
| 13. | Non-participation | How many people refused to participate or dropped out? Reasons? Data for ‘refusals’ were not available. Non-response to the recruitment email and total recruitment sample is included in the Results section. |
| Setting |  |  |
| 14. | Setting of data collection | Where was the data collected? e*.g. home, clinic, workplace*  The interviews were conducted via telephone. |
| 15. | Presence of non-participants | Was anyone else present besides the participants and researchers?  Not necessary for telephone interviews. |
| 16. | Description of sample | What are the important characteristics of the sample? *e.g. demographic data, date*  We did not collect gender/age/race/etc. We do report roles in the participant table. |
| Data collection |  | See notes for each item |
| 17. | Interview guide | Were questions, prompts, guides provided by the authors? Was it pilot tested? Interview questions were not provided to participants. We did not pilot the Interview Guide. Interview guide questions are included the manuscript. |
| 18. | Repeat interviews | Were repeat interviews carried out? If yes, how many? N/A |
| 19. | Audio/visual recording | Did the research use audio or visual recording to collect the data? Noted in the Analysis section |
| 20. | Field notes | Were field notes made during and/or after the interview or focus group? No field notes were taken during the interviews. Issues impacting data collection, quality, or analysis (e.g., concerns with participant comprehension, distractions, audio quality, etc.) would have been noted and recorded, but none were encountered. |
| 21. | Duration | What was the duration of the interviews or focus group?  Included |
| 22. | Data saturation | Was data saturation discussed?  No. We described in the Participant and Results sections that this was a convenience sample and we conducted interviews with 100% of available participants, so data saturation was not applied to our data collection approach. |
| 23. | Transcripts returned | Were transcripts returned to participants for comment and/or correction? We did not conduct participant transcript review. |
| **Domain 3: analysis and findings**z |  |  |
| Data analysis |  | See notes for each item |
| 24. | Number of data coders | How many data coders coded the data? Included in Analysis section |
| 25. | Description of the coding tree | Did authors provide a description of the coding tree? Consistent with Clarke & Braun’s thematic analysis approach, we did not use hierarchical coding. Single-level codes were clustered in categories for thematic identification and development, so no coding tree was generated. |
| 26. | Derivation of themes | Were themes identified in advance or derived from the data? Included in Analysis section |
| 27. | Software | What software, if applicable, was used to manage the data? Included in Analysis section |
| 28. | Participant checking | Did participants provide feedback on the findings? Member-checking did not occur. |
| Reporting |  | All these below are in manuscript. |
| 29. | Quotations presented | Were participant quotations presented to illustrate the themes / findings? Was each quotation identified? e*.g. participant number* |
| 30. | Data and findings consistent | Was there consistency between the data presented and the findings? |
| 31. | Clarity of major themes | Were major themes clearly presented in the findings? |
| 32. | Clarity of minor themes | Is there a description of diverse cases or discussion of minor themes? |
